# Transdermal Delivery of Small Interfering RNA with Elastic Cationic Liposomes in Mice

**DOI:** 10.1155/2013/149695

**Published:** 2013-12-26

**Authors:** Yoshiyuki Hattori, Masataka Date, Shohei Arai, Kumi Kawano, Etsuo Yonemochi, Yoshie Maitani

**Affiliations:** Institute of Medicinal Chemistry, Hoshi University, Ebara 2-4-41, Shinagawa-ku, Tokyo 142-8501, Japan

## Abstract

We developed elastic cationic liposomal vectors for transdermal siRNA delivery. These liposomes were prepared with 1,2-dioleoyl-3-trimethylammonium-propane (DOTAP) as a cationic lipid and sodium cholate (NaChol) or Tween 80 as an edge activator. When NaChol or Tween 80 was included at 5, 10, and 15% (w/w) into DOTAP liposomal formulations (C5-, C10-, and C15-liposomes and T5-, T10-, and T15-liposomes), C15- and T10-liposomes showed 2.4- and 2.7-fold-higher elasticities than DOTAP liposome, respectively. Although the sizes of all elastic liposomes prepared in this study were about 80–90 nm, the sizes of C5-, C10- and C15-liposome/siRNA complexes (lipoplexes) were about 1,700–1,800 nm, and those of T5-, T10-, and T15-lipoplexes were about 550–780 nm. Their elastic lipoplexes showed strong gene suppression by siRNA without cytotoxicity when transfected into human cervical carcinoma SiHa cells. Following skin application of the fluorescence-labeled lipoplexes in mice, among the elastic lipoplexes, C15- and T5-lipoplexes showed effective penetration of siRNA into skin, compared with DOTAP lipoplex and free siRNA solution. These data suggest that elastic cationic liposomes containing an appropriate amount of NaChol or Tween 80 as an edge activator could deliver siRNA transdermally.

## 1. Introduction

RNA interference (RNAi) offers potential for treating a wide variety of disorders through selective silencing of disease-relevant RNAs [1, 2]. Topical use of small interfering RNA (siRNA) has been increasingly studied for its applicability in treating skin disease [3]. However, transdermal naked siRNA delivery is limited due to its low permeability of skin barriers such as the stratum corneum and the epidermal layer.

Liposomes have been widely investigated in dermatology as transdermal carrier systems [4]. Of particular interest are cationic liposomes for passive transdermal delivery of siRNA, since their opposite charges spontaneously result in complexation due to electrostatic interactions. Cationic liposomes protect siRNA from degradation and allow the introduction of siRNA into cells. It has been reported that elastic cationic liposomes (ultradeformable cationic liposomes) can penetrate intact skin while carrying plasmid DNA (pDNA) [5] and antisense oligonucleotides [6] when applied under nonoccluded conditions. Elastic cationic liposomes consist of a cationic lipid and an edge activator that is responsible for the elasticity of the membrane. An edge activator is usually a single-chain surfactant that destabilizes the lipid bilayers of the liposomes and increases the deformability of the bilayers [7]. For transdermal gene delivery, sodium cholate (NaChol), sodium deoxycholate (NaDChol), and Tween 80 were used as an edge activator [5, 6, 8]; however, there is little information about the feasibility and effect of an edge activator on elastic liposomes for transdermal siRNA delivery *in vivo*.

Deformability of elastic liposome enables therapeutic siRNA to penetrate the skin barrier by the stratum corneum. A cationic lipid, 1,2-dioleoyl-3-trimethylammonium propane (DOTAP), has frequently been used as a cationic lipid for a liposomal delivery system of siRNA by several research groups [9–12]. Moreover, we previously reported that NaChol and Tween 80 incorporated into liposomal membranes confer deformation and flexibility to ultradeformable liposomes [13, 14]. Therefore, in the present study, for transdermal siRNA delivery, we optimized the formulation of elastic DOTAP liposomes including NaChol or Tween 80, which are positively charged liposomes exhibiting some deformability, and evaluated the permeability of siRNA in mouse skin after topical application of elastic liposome/siRNA complex (elastic lipoplex).

## 2. Materials and Methods

### 2.1. Materials

1,2-Dioleoyl-3-trimethylammonium propane methyl sulfate (DOTAP) was purchased from Avanti Polar Lipids Inc. (Albaster, AL, USA). Sodium cholate hydrate (NaChol) was purchased from Sigma-Aldrich Japan (Tokyo, Japan). Tween 80 was purchased from NOF Inc. (Tokyo, Japan). Lissamine rhodamine B 1,2-dihexadecanoyl-sn-glycero-3-phosphoethanolamine, triethylammonium salt (rhodamine-DHPE), was purchased from Invitrogen (Carlsbad, CA, USA). All other chemicals were of the finest grade available.

### 2.2. siRNA

The siRNAs targeting nucleotides of *firefly* luciferase (Luc siRNA) and nonsilencing siRNA (Cont siRNA) as a negative control were synthesized by Sigma Genosys (Tokyo, Japan) [15]. The siRNA sequences of the Luc siRNA were passenger strand: 5′-CCGUGGUGUUCGUGUCUAAGA-3′ and guide strand: 5′-UUAGACACGAACACCACGGUA-3. The siRNA sequences of the Cont siRNA were passenger strand: 5′-CCGUACUAGCCAUUAUGCGUC-3′ and guide strand: 5′-CGCAUAAUGGCUAGUACGGGU-3′. 6-Carboxyfluorescein-aminohexyl-amidite- (FAM-) labeled luciferase (pGL3) siRNA was obtained from Cosmo Bio Co., Ltd. (Tokyo, Japan). The siRNA sequences of the FAM-labeled luciferase siRNA were passenger strand: 5′-UCGAAGUACUCAGCGUAAGdTdT-3′ and guide strand: 5′-CUUACGCUGAGUACUUCGAdTdT-3′.

### 2.3. Preparation of Liposomes and Lipoplexes

NaChol and Tween 80 were dissolved in phosphate-buffered saline (PBS, pH 7.4) by vortexing. DOTAP liposomes were prepared by vortexing in PBS with a concentration of 10 mg/mL DOTAP until a milky suspension was obtained as previously reported [6]. Elastic liposomes were prepared by vortexing after adding NaChol or Tween 80 solution to DOTAP solution at final concentrations of 5, 10, and 15% (w/w) (Table 1). For preparation of rhodamine-labeled liposomes, rhodamine-DHPE was incorporated into the liposomal formulation at 0.2 mol% in the total lipid. The liposomes were sonicated in a bath-type sonicator (Branson 25010J-MTH, USA) for 1 h at room temperature and then filtered 31 times through 100 nm polycarbonate membrane filters (Whatman, Brentfort, UK).

Lipoplexes were prepared by mixing the liposome with siRNA at a weight ratio of DOTAP/siRNA of 14/1 (charge ratio (+/−) of 6/1), as previously reported [9], and then incubated at room temperature for 10–15 min. Average diameters and *ζ*-potentials of liposomes and lipoplexes were measured by dynamic light-scattering and electrophoresis light-scattering methods, respectively (ELS-Z2; Otsuka Electronics, Osaka, Japan). All measurements were performed at 25 ± 1°C, after diluting the liposome and lipoplex suspension with Milli-Q water.

### 2.4. Measurement of Elasticity

The elasticity of the bilayer of liposomes was directly proportional to *J*_flux_ × (*r*_*v*_/*r*_*p*_)^2^:

(1) $$\begin{array}{c} \text{Elasticity}\;\left(\mu \text{g}\cdot {\text{sec}}^{-1}\cdot \text{c}{\text{m}}^{-2}\right)=J_{\text{flux}}\times {\left(\frac{r_{v}}{r_{p}}\right)}^{2}, \end{array}$$

where *J*_flux_ is the rate of penetration through a permeability barrier, *r*_*v*_ is the size of liposomes after extrusion, and *r*_*p*_ is the pore size of the barrier:

(2) Jflux(μg·sec−1·cm−2) =J(DOTAP(μg))(extrusion  time(sec)×membrane  area(cm2)).

To measure *J*, the liposomes were extruded through a polycarbonate membrane (4.9 cm^2^)with a pore diameter of 50 nm (*r*_*p*_), at a pressure of 0.5 MPa. After 10 min of extrusion, the extrudate was weighed, and then concentration of DOTAP in the extrudate was calculated by measurement of absorbance at 228 nm after the addition of equal volume of methanol. The average liposome diameter after extrusion (*r*_*v*_) was measured by ELS-Z2 as described in the above section.

### 2.5. Cell Culture

Human cervical carcinoma SiHa cells stably expressing *firefly* luciferase (FL-SiHa) were donated by Dr. Kenji Yamato (Department of Gastroenterology, Tsukuba University, Tsukuba, Japan). FL-SiHa cells were grown in Eagle's MEM, supplemented with 10% heat-inactivated fetal bovine serum (FBS), 100 *μ*g/mL kanamycin, and 1 mg/mL G418 at 37°C in a 5% CO_2_ humidified atmosphere.

### 2.6. Luciferase Activity

FL-SiHa cells were seeded at a density of 4 × 10^5^ cells per well in 24-well plates and maintained for 24 h before transfection. For transfection, each lipoplex of siRNA was diluted with serum-free medium to a final concentration of 50 nM Luc or Cont siRNA and then gently added to the cells. After incubation for 3 h at 37°C, the cells were added with FBS to a final concentration of 10% and then incubated for another 45 h. Lipofectamine RNAiMax lipoplex (Invitrogen Corp.) was prepared according to the manufacturer's protocol. Forty-eight hours after the transfection, luciferase activity was measured as counts per sec (cps)/*μ*g protein using the luciferase assay system (Promega, Madison, USA) and BCA reagent (Pierce, Rockford, IL, USA), as previously reported [15].

### 2.7. Cytotoxicity

FL-SiHa cells were seeded at a density of 6 × 10^4^cells per well in 96-well plates and maintained for 24 h before transfection. For transfection, each lipoplex of siRNA was diluted with serum-free medium to a final concentration of 50 nM Cont siRNA and then gently added to the cells. After incubation for 3 h at 37°C, the cells were supplemented with FBS to a final concentration of 10%. After 45 h of incubation, the medium was removed, and the cells were treated with WST-8 (2-(2-methoxy-4-nitrophenyl)-3-(4-nitrophenyl)-5-(2,4-disulfophenyl)-2H-tetrazolium, monosodium salt) solution (10 *μ*L) in medium containing serum (100 *μ*L) for 30 min. Cell viability is expressed relative to the absorbance at 450 nm of untransfected cells.

### 2.8. Skin Penetration of Liposomes and Lipoplexes

The excised skin of hairless mice (Laboskin, HOS: HR-1 male, 7 weeks, Hosino Laboratory Animals, Inc., Ibaraki, Japan) was used for the investigation of skin permeability by elastic liposomes. For this purpose, rhodamine-labeled liposomes were nonocclusively applied on the Laboskin (3.14 cm^2^) for 6 h.

Female HR-1/Hos hairless mice (6 weeks of age) were purchased from Hosino Laboratory Animals, Inc. All *in vivo* experiments were approved by the Institutional Animal Care and Use Committee of Hoshi University. For observation of the skin penetration of the elastic lipoplexes, the lipoplexes of rhodamine-labeled liposomes and FAM-labeled luciferase siRNA (50 *μ*g) were applied nonocclusively on the dorsal skin (3.14 cm^2^) under anesthesia for 6 h. The mice were kept on a hot plate during the sedation period.

After application with elastic liposomes or lipoplexes, the skins were embedded in OCT compound (Tissue-Tek, Sakura Finetechnical Co., Ltd., Tokyo, Japan) and processed by frozen sectioning at 20 *μ*m. Each frozen section was mounted on a silane-coating slide (Muto Pure Chemicals Co., Ltd., Tokyo, Japan). Localizations of FAM-labeled siRNA and rhodamine-labeled liposomes were examined using an LSM5 EXCITER confocal laser scanning microscope (Carl Zeiss, Thornwood, NY, USA). FAM-labeled siRNA was imaged using an argon laser at 488 nm excitation, and fluorescence emission was observed with a filter, BP505-530. For rhodamine-labeled liposome, maximal excitation was performed with a 543 nm internal He-Ne laser, and fluorescence emission was observed with an LP560.

### 2.9. Statistical Analysis

The statistical significance of differences between mean values was determined by using Student's *t*-test. A *P* value of 0.05 or less was considered significant.

## 3. Results and Discussion

Transdermal delivery of naked siRNA is limited due to its low stability in skin and low permeability by various skin barriers such as the stratum corneum and epidermal layer. In this study, we investigated whether cationic elastic liposomes could effectively deliver siRNA transdermally into the skin. Here, we used six formulae for elastic liposomes consisting of DOTAP as a cationic lipid and NaChol or Tween 80 as an edge activator (Table 1): DOTAP liposomes containing Tween 80 at 5, 10, and 15% (w/w) (T5-, T10-, and T15-liposomes) and DOTAP liposomes containing NaChol at 5, 10, and 15% (w/w) (C5-, C10-, and C15-liposomes). DOTAP liposomes without an edge activator were used as a control.

Average diameter and *ζ*-potential of DOTAP liposome were about 110 nm and 40 mV, respectively (Table 1). The addition of NaChol to the formulation of DOTAP liposome decreased the size after preparation of elastic liposome and increased the *ζ*-potential. C5-, C10-, and C15-liposomes were 80–90 nm in size and had *ζ*-potential of 42–52 mV. On the other hand, the addition of Tween 80 decreased both the size and the *ζ*-potential, and T5-, T10-, and T15-liposomes were about 80–90 nm in size and had *ζ*-potential of about 26–36 mV.

**Figure 1:**
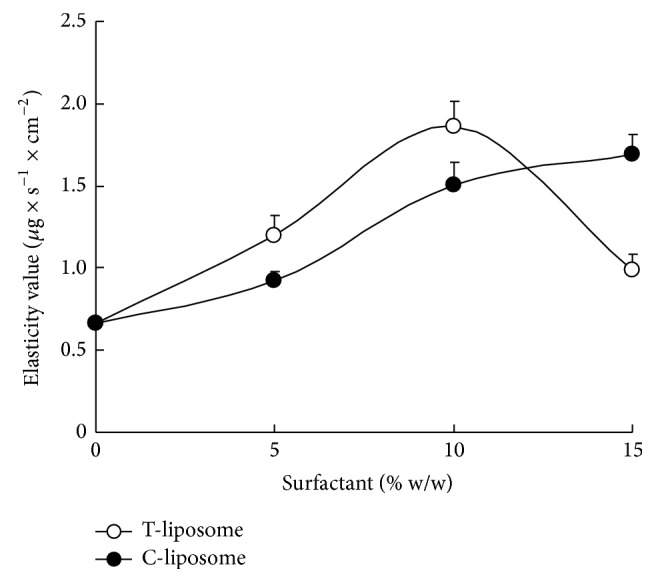
Effect of Tween 80 or NaChol content (w/w) on elasticity values of cationic elastic liposomes. Elasticity values were calculated by *J*_flux_ × (*r*_*v*_/*r*_*p*_)^2^. Each value represents the mean ± S.D. (*n* = 3).

Next, we confirmed the deformability of the elastic liposomes. As shown in Figure 1, the elasticity of liposomes containing Tween 80 peaked at 10% (w/w) Tween 80 content and was reduced at 15% (w/w). T10-liposomes showed 2.7-fold-higher elasticity than DOTAP liposomes. In contrast, the elasticity of liposomes containing NaChol was increased with an increase of NaChol content, and C15-liposome showed 2.4-fold-higher elasticity than DOTAP liposome. These findings indicate that the optimal amount of edge activator for elasticity of DOTAP liposome differed between the liposomes containing NaChol and Tween 80.

In the following step, we prepared elastic lipoplex by mixing the liposomes with siRNA. The sizes of DOTAP, C5-, C10-, and C15-lipoplexes were about 1,700–1,860 nm, and those of T5-, T10-, and T15-lipoplexes were about 550–780 nm. Notably, the addition of Tween 80 was observed to reduce the size of the lipoplexes. This might be due to effect of polyoxyethylene on the surface of elastic lipoplex containing Tween 80.

**Figure 2:**
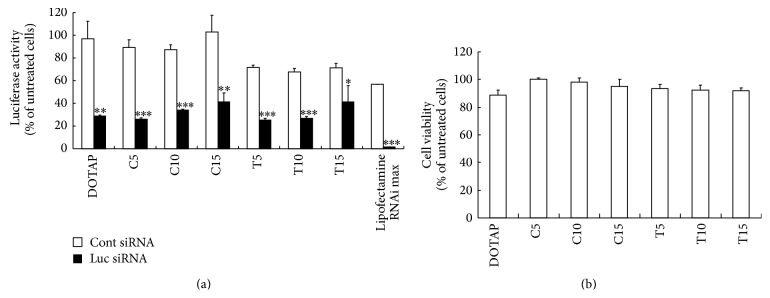
The suppression of luciferase activity (a) and cytotoxicity (b) after siRNA transfection by elastic cationic liposomes. In (a), cationic lipoplexes were added to FL-SiHa cells at 50 nM siRNA. The luciferase assay was carried out 48 h after incubation of the lipoplexes. Statistical significance was evaluated by Student's *t*-test. ^*^ *P* < 0.05, ^**^ *P* < 0.01, and ^***^ *P* < 0.001, compared with lipoplex of Cont siRNA. In (b), cytotoxicity was evaluated 48 h after transfection. In (a) and (b), each column represents the mean ± S.D. (*n* = 3).

Next, we investigated the silencing effects of luciferase mRNA and cytotoxicity in SiHa-Luc cells after transfection of the lipoplexes with Luc siRNA (Figures 2(a) and 2(b)). Although all formulations showed the suppression of luciferase activity without cytotoxicity, an increase of Tween 80 content (T15-liposome) decreased the silencing effect by elastic lipoplex. As a control, lipofectamine RNAiMax, a commercially available transfection reagent, induced strong suppression by siRNA transfection.

**Figure 3:**
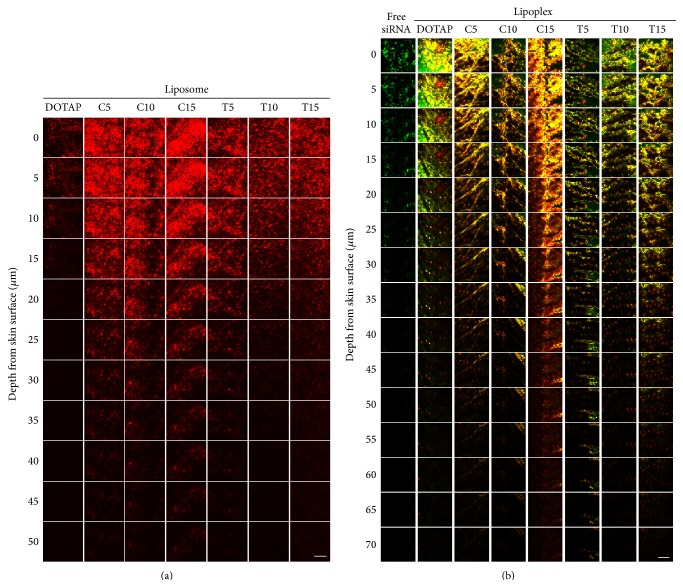
Permeability of rhodamine-labeled liposomes (a) and lipoplexes of FAM-labeled siRNA and rhodamine-labeled liposome (b) into mouse skin after topical application for 6 h. In (a), the localizations of rhodamine-labeled liposome are shown as red signals. In (b), the localizations of FAM-labeled siRNA and rhodamine-labeled liposome are shown as green and red signals, respectively. Scale bar = 200 *μ*m.

To examine the skin permeability of elastic liposomes, we applied rhodamine-labeled elastic liposomes on excised mouse skin for 6 h and observed their localization by confocal microscopy.  Figure 3(a) shows horizontal sections of mouse skin on which the liposomes were applied. The photographs represent sliced images of mouse skin from the surface and up to a thickness of 50 *μ*m. When rhodamine-labeled DOTAP liposome was applied on the skin, the fluorescence was observed until a thickness of 15 *μ*m (Figure 3(a)). However, the liposomes containing NaChol improved skin penetration with an increase in NaChol content, and fluorescence in the skin after application with C15-liposome was strongly observed up to a depth of 50 *μ*m. Furthermore, the liposomes containing Tween 80 also showed increased skin penetration compared with DOTAP liposomes, but an increase of Tween 80 content decreased skin penetration. Fluorescence in skin after the application of T5-, T10-, and T15-liposomes was observed at thicknesses of 45, 30, and 35 *μ*m, respectively. These findings indicate that C15- and T5-liposomes could deeply penetrate into mouse skin.

Finally, we applied the elastic lipoplexes on mouse skin (Figure 3(b)). The photographs represent sliced images of mouse skin from the surface and up to a thickness of 70 *μ*m. In mouse skin on which naked FAM-labeled siRNA solution was applied, the fluorescence was weakly observed at around 25 *μ*m in thickness. In contrast, elastic lipoplexes containing NaChol showed high skin penetration of both siRNA and liposomes with an increase of NaChol content, and the fluorescence of FAM-labeled siRNA and rhodamine-labeled liposomes after application with C15-lipoplex was observed at a thickness of around 70 *μ*m although the size of C15-lipoplex was large (about 1.8 *μ*m). Moreover, the elastic lipoplex containing Tween 80 also showed improvement of skin penetration. Among the lipoplexes containing Tween 80, T5-lipoplex (600 nm) showed improved skin penetration, and the fluorescence of both siRNA and liposomes was observed at around a depth of 70 *μ*m. The fluorescence of FAM-labeled siRNA after application of C15- and T5-lipoplexes largely merged with that of rhodamine-labeled C15- and T5-liposomes, respectively, indicating that siRNA penetrated into the skin as a lipoplex. From these findings, the elastic liposomes might squeeze between cells in the stratum corneum and penetrate the intact skin *in vivo* through a transcutaneous hydration gradient because the elastic liposome was shown to have high stress-dependent adaptability [16]. In the liposomes containing NaChol, C15-liposome and lipoplex showed the highest elasticity and skin penetration, respectively; in contrast, in the liposomes containing Tween 80, T10-liposome showed the highest elasticity, but T5-lipoplex showed the highest skin penetration, indicating that the optimal amount of Tween 80 for elasticity and penetration might differ between liposome and lipoplex although we did not measure the elasticity of elastic lipoplex.

In pDNA delivery, NaChol, NaDChol, and Tween 80 have often been used as an edge activator of elastic liposomes. Ultradeformable neutral liposome composed of egg phosphatidylcholine (ePC)/NaChol or ePC/NaDChol displayed the highest level of *in vivo* transdermal pDNA absorption [8]. Ultradeformable cationic liposomes composed of DOTAP/DOPE/NaDChol [5] and DOTAP/NaChol [6] have been used for the topical delivery of pDNA and antisense oligonucleotide, respectively. However, there is little information about the effect of an edge activator on elastic liposomes for transdermal siRNA delivery *in vivo*. Geusens et al. reported that ultradeformable cationic liposomes composed of DOTAP and NaChol at a ratio of 6/1 (w/w) (about 14% (w/w)) showed a high gene knock-down effect in melanoma cells by siRNA [9]; however, in *in vivo* siRNA transfer, they reported that SECosome (surfactant-ethanol-cholesterol) consisted of DOTAP, NaChol, and cholesterol at a ratio of 6/1/1 (w/w/w) in 30% ethanol, penetrated into epidermis of excised human skin, and suggested that the combination of ethanol and sodium cholate was needed for effective penetration of lipoplexes into skin [17]. In this study, we prepared elastic liposomes containing NaChol or Tween 80 as an edge activator and found that C15- and T5-lipoplexes of siRNA showed high *in vitro* gene suppression and skin penetration. These findings suggested that C15- and T5-liposomes are outstanding tools for transdermal siRNA delivery in mouse skin.

## 4. Conclusion

In this study, we could develop elastic cationic liposomes for siRNA skin delivery. Our results suggest that 15% (w/w) NaChol or 5% (w/w) Tween80 is the most effective formulation of DOTAP-based liposome for transdermal siRNA delivery. Further study should be performed to examine the effect of gene expression after *in vivo* siRNA application.

## Figures and Tables

**Table 1:** Particle size and *ζ*-potential of liposomes and lipoplexes used in this study.

Vesicles	% (w/w)			Liposome^a^			Lipoplex^a,b^	
	DOTAP	NaChol^c^	Tween 80	Size (nm)	PD^d^	*ζ*-potential (mV)	Size (nm)	PD^d^
DOTAP	100	—	—	109 ± 2	0.10 ± 0.02	40.2 ± 2.4	1724 ± 123	0.63 ± 0.04
C5	95	5	—	84 ± 2	0.11 ± 0.02	42.4 ± 1.4	1795 ± 432	0.65 ± 0.13
C10	90	10	—	87 ± 3	0.16 ± 0.00	52.2 ± 2.2	1809 ± 161	0.61 ± 0.05
C15	85	15	—	82 ± 1	0.13 ± 0.01	47.8 ± 1.7	1859 ± 115	0.56 ± 0.02
T5	95	—	5	82 ± 1	0.19 ± 0.01	36.0 ± 0.8	582 ± 138	0.26 ± 0.04
T10	90	—	10	87 ± 1	0.17 ± 0.01	26.4 ± 1.8	550 ± 45	0.26 ± 0.01
T15	85	—	15	83 ± 2	0.15 ± 0.01	30.8 ± 1.5	775 ± 106	0.33 ± 0.04

^a^In water. ^b^Weight ratio of lipid/siRNA = 14/1. ^c^NaChol, sodium cholate. ^d^PD, polydispersity index. Values represent means ± S.D. (*n* = 3).
